# Medical education today: all that glitters is not gold

**DOI:** 10.1186/s12909-019-1535-9

**Published:** 2019-04-16

**Authors:** L. Maximilian Buja

**Affiliations:** Department of Pathology and Laboratory Medicine, McGovern Medical School, The University of Texas Health Science Center at Houston (UTHealth), 6431 Fannin St., MSB2.276, Houston, TX 77005 USA

**Keywords:** Undergraduate medical education, Graduate medical education, Medical science, pathology, physician-scientists.

## Abstract

**Background:**

The medical education system based on principles advocated by Flexner and Osler has produced generations of scientifically grounded and clinically skilled physicians whose collective experiences and contributions have served medicine and patients well. Yet sweeping changes launched around the turn of the millennium have constituted a revolution in medical education. In this article, a critique is presented of the new undergraduate medical education (UME) curricula in relationship to graduate medical education (GME) and clinical practice.

**Discussion:**

Medical education has changed and will continue to change in response to scientific advances and societal needs. However, enthusiasm for reform needs to be tempered by a more measured approach to avoid unintended consequences. Movement from novice to master in medicine cannot be rushed. An argument is made for a shoring up of biomedical science in revised curricula with the beneficiaries being nascent practitioners, developing physician-scientists --and the public.

**Conclusion:**

Unless there is further modification, the new integrated curricula are at risk of produce graduates deficient in the characteristics that have set physicians apart from other healthcare professionals, namely high-level clinical expertise based on a deep grounding in biomedical science and understanding of the pathologic basis of disease. The challenges for education of the best possible physicians are great but the benefits to medicine and society are enormous.

## Background

### Introduction

The traditional medical education system widely adopted throughout most of the twentieth century has produced generations of scientifically grounded and clinically skilled physicians who have served medicine and society well. Yet sweeping changes launched around the turn of the millennium have constituted a revolution in undergraduate medical education (UME) and graduate medical education (GME) [1–3]. While continual assessment leading to measured adaptation is essential for the enduring value of a system, simultaneous and multifaceted change such as that occurring in the traditional medical education system qualifies as disruptive innovation [4]. The purpose of this article is to offer a critique and express a major concern by a physician-scientist, pathologist and medical educator that the contemporary medical education system is being subject to the downside of disruptive innovation with unintended and potentially detrimental long-term outcomes for academic medicine and clinical practice.

### The past century in medical education

The education of a physician has developed to encompass pre-medical preparation, a course of study in a medical school which is typically a major component of an academic medical center (AHC), and medical specialty training in residency and fellowship programs, UME and GME, respectively [5, 6]. This education provides the basis for a professional career enhanced by continuing medical education and life-long learning. Early in the twentieth century, medical education became guided by principles articulated by Abraham Flexner and William Osler. Flexner recommended that medical schools should be university based, have minimum admission requirements, implement a rigorous curriculum with applied laboratory and clinical science content, and have faculty actively engaged in research [5, 7]. Osler championed bedside teaching, bringing medical students into direct contact with patients, and learning medicine from these direct experiences under the guidance of faculty clinicians [7, 8]. The result was the establishment of two key components or pillars of medical education, namely, the basic or foundational sciences and the clinical sciences [2]. The two-pillar model of medical education provided the conceptual basis for a four-year UME curriculum comprising biomedical science courses in the pre-clinical years and clinical clerkships in the clinical years. Medical schools utilizing this construct produced scientifically grounded physicians capable of a high level of clinical practice as well as a subset who pursued highly successful careers as physician-scientists and academicians [9].

### AHCs and healthcare system

A fundamental element in the achievement of medical schools in the twentieth century was the development of medical education as a public trust and social contract between the medical schools and society [5]. However, in-depth analysis of the history of medical education has shown that it is inextricably intertwined with healthcare delivery and broader societal norms [5–7]. UME and much of GME take place in academic health centers (AHC), which must function in the world of healthcare delivery [10], and are subject to the complexities of the associated health care system in which they operate, including the fragmented American healthcare system [11–14].

### Calls for curriculum reform and restructuring

In this context, discontent among academics and professional organizations concerning the traditional medical education construct has accelerated in recent years [1–3, 15–21]. Both the teaching methodology and the content of the established curriculum have come under severe criticism. Calls have been made repeatedly for the cultivation of a different type of physician more attuned to and equipped for practice in the current healthcare scene [15–20].

## Discussion

### Reform movement and integrated curriculum

To promote more active learning and less passive learning, curriculum developers have introduced a variety of approaches, including small group sessions, problem-based learning, self-directed learning, team-based learning, and flipped classrooms as replacements for the traditional lecture format [21]. However, many in the reform movement consider that pedagogical reform, while necessary, must be joined by content reform to develop the requisite skill set in future practitioners of medicine [15–20]. As a result, there has been a movement in mass toward adoption of a radically redesigned curriculum as a third wave, post-Flexnerian approach to medical education [1, 2]. A major goal of the curriculum reformers is to produce physicians who can deliver an individualized plan of care that reflects the physician’s mastery of basic physiology, awareness of the best current evidence, skillful patient communication, and shared decision-making [20].

The ideal of the post-Flexnerian third wave is a fully integrated curriculum in place of the traditional curriculum comprised of a distinct pre-clinical component with subject-based courses and a subsequent clinical component [22]. Initial implementation involves partial integration comprising horizontal integration defined as integration across disciplines but within a finite period of time and vertical integration representing integration across time with breakdown of the traditional barrier between basic and clinical sciences. A fully integrated curriculum is characterized by spiral integration encompassing both horizontal and vertical integration combining integration across time and across disciplines [22].

This revised design also includes added content addressing broader issues constituting “Health Systems Science” as a third pillar of medical education co-equal with basic and clinical medical sciences [23–26]. Topics include population health, health policy, healthcare delivery systems, and interdisciplinary care. A correlate is the replacement of the biomedical model of health and disease with a broader biopsychosocial model of health, disease and the patient-physician relationship [23, 27].

A related development is the implementation of the new MCAT that aims to balance testing in the natural sciences with testing in the social and behavioral sciences and assessing critical analysis and reasoning skills. The redesign is based on the premise that tomorrow’s physicians need broader skills and knowledge than in the past [28]. Medical education reform also includes heavy emphasis on professionalism and professional identity development [29]. The reforms also are aimed at achieving a more coherent continuum of medical education [30]. My institution, McGovern Medical School of The University of Texas Health Science Center at Houston, embarked on the path of curriculum restructuring in 2013 and has instituted such a redesigned curriculum beginning in 2016 [31].

### Influence of oversight bodies

Advances in medical care and technology have been driving forces behind these curriculum changes. In the United States, a major impetus for such curriculum changes has come from the Liaison Committee for Medical Education (LCME), and its sponsoring institutions, the American Association of Medical Colleges (AAMC) and the American Medical Association (AMA), and the Accreditation Council for Graduate Medical Education (ACGME) (more accurately, thought leaders in these organizations) [32]. Regulatory bodies in other countries have had similar roles [22]. Curriculum reformers have used the imperative of actual and perceived expectations of the LCME as a driver of curriculum revision.

### Characteristics of Today’s medical students

A major consideration in any discussion of education is the profile of the students. Analysis of today’s students is that they score higher on assertiveness, self-liking, narcissistic traits, high expectations, and some measures of stress, anxiety and poor mental health, and also lower on self-reliance [33–35]. These generational characteristics are rooted in shifts in culture and reflect changes in society. These character traits are clearly established by the time students enter medical school.

Notable individual exceptions reinforce the average characteristics of today’s students which have definite positive aspects, such as the focus on the individual, but also some negative consequences [33–35]. Motivation can become dysfunctional so that high levels of dedication to a previously enjoyed activity can result in burnout. Burnout is alarmingly high among today’s medical students and residents [36, 37]. Burnout is a psychosocial syndrome that is associated with motivational, performance and psychological difficulties. Perfectionism, defined as a combination of high standards and high self-criticism, is also on the rise [38, 39]. The two may compound each other.

The characteristics of today’s medical students including their strengths and vulnerabilities, present special challenges for faculty engaged in their education [40–43]. Notably, while these students have high I.Q.s, they typically show little desire to read long texts [33]. The implication for educational design (pedagogy) is that these students likely benefit from a structured but also more interactive learning experience and that instruction may need to be delivered in shorter segments and perhaps incorporate more material in media such as videos and an interactive format. But, even when the classroom hour is used for so-called active learning approaches, such as the flipped classroom, attendance is still often poor. There has been a proliferation of commercial products, including First Aid, Firecracker, Osmosis and Pathoma, that attract students with shortcut approaches, including flashcards and videos, for passing standardized tests [44]. These products cater to the study habits of many of today’s students. Many of today’s medical students are opting for elective perusal online of previously recorded lectures and the use of various previously mentioned study aids while minimizing direct classroom interaction with professors [45].

### General critique

While apparently accepting the practices of today’s medical students as a *fait accompli*, a key tenant of the reform movement is that the traditional subject-based and lecture-based curriculum has failed to accomplish the desired outcome of producing physicians for the twenty-first century [20]. Content reformers favor a repeal of major parts of the traditional UME curriculum to make room for the lessons that are aimed at allowing students to develop skills in modern clinical reasoning and decision-making. Major goals of integration are to break down barriers between the basic and clinical sciences and to promote retention of knowledge and acquisition of skills through repetitive and progressive development of concepts and their applications [22].

Reformers recognize that implementation of the new curriculum requires trade-offs and hard choices. They have clearly articulated that topics such as clinical decision-making, comparative effectiveness and other Health Systems Science topics must take priority over the depth of basic science content presented in traditional courses [20]. The argument is made that major revamping of basic science in the curriculum is acceptable because of perceived major overlap and repetition among traditional basic science courses. There also is the often unstated but implied view that traditional basic science courses burden medical students with excessive and unnecessary detail. While strong emphasis is placed on integrating basic science courses and providing clinical experiences early in the curriculum, the extension of basic science content into the clinical years has been identified as a major challenge and a major shortcoming of integrated curricula [22, 46].

The first two years of the UME curriculum is the only time in the entire professional career of a physician when the fundamentals of biomedical science and the clinical skills of history taking and physical examination intersect coherently, and are formally taught and learned. A background in factual knowledge and relationships among facts is crucial for critical thinking and evidence-based decision-making in medicine [46–49]. Studies have shown that factual knowledge of medical science is essential for the development of clinical skill [46–50]. Clinical knowledge is gained from the integration of conceptual knowledge (facts, “what” information), strategic knowledge (“how” information) and conditional knowledge (“why” information) [49]. There is no short cut here; a certain amount of memorization and with some repetition is required. It is counterproductive to dilute the learning experience of the core material in the pre-clinical years by substituting other topics that are best learned after a foundation is laid and its strength tested through the crucible of clinical practice.

### Competency-based education: time-based versus competency-based medical education and accelerated medical education

Momentum has continued to grow for demonstration of a set of competencies rather than cognitive knowledge alone as the primary outcome of UME as well as GME. The movement toward outcomes and competency-based education in UME was presaged by a focus on innovation in GME, which led to the introduction by the Accreditation Council for Graduate Medical Education (ACGME) of the six competencies as key elements in residency training programs [51, 52]. Change in the world of GME was compounded by the introduction of the duty hour requirements at about the same time [53]. The ACGME has moved further along the path of competency-based training with the introduction of milestones as a focus of the new accreditation system (NAS) [54, 55]. Competencies also have been linked to Entrustable Professional Activities [56].

Some are taking the competency construct further by promoting time variable criteria for the granting of the medical degree as well as certification in medical specialties following a period of graduate training [57–62]. Others are promoting an accelerated three-year UME program [63].

### General critique

All would agree that the goal of medical education is to produce competent physicians. However, the educational approach embodied in competence-based curricula for highly skilled professions including medicine versus lower level occupations has been found to be philosophically questionable, methodologically complex and highly controversial [64, 65]. The logistics of implementing such programs are daunting and represent another major draw on faculty time to provide evaluation of the ascertainment of the set of competencies and entrustable professional activities (EPAs) of the learners [56, 66]. A more feasible approach would be to maintain fixed time programs but allow accelerated advancement coupled with opportunities for dual degrees, pursuit of research, and other projects [67].

Arguments in favor of reduction of UME to a 3 year program include increased production of physicians to meet the shortage and reduction of student debt. The current interest in some quarters for a 3 year program represents the third time in the last century this idea has been promoted [64]. This third wave will have to face many of the same issues that affected the previous two attempts.

### Impact of student evaluation systems

How students function in an educational program is inextricably linked to how they are evaluated. Recurrent movements to abolish grades, exams and honor societies to mitigate undue competiveness, stress and general malaise is the present educational *zeitgeist* [68–72]. For many years, the standard system of student evaluation was based on numerical grades in every course and led to a cumulative numerical score and class ranking. As a component of disruptive innovation, some medical schools have completely abolished grades and implemented pass-fail systems. However, most medical schools, including some who have tried the purely pass-fail approach, have arrived at a system of Honors, High Pass, Pass, Marginal Pass and Fail -- essentially the A through F system used in K-12 education [73].

This has led to the rise of the exaggerated importance of United States Medical Licensing Exam (USMLE) scores, particularly, USMLE Step 1 scores, as the major or sole objective evaluation of cognitive achievement of medical students. Proponents argue that the new curricula are successful because students are performing at least as well on USMLE Step 1 as they did in the old curricula, and that they do as well in pass-fail systems as in systems with grades [68–72]. However, these advocates, in essence, are contributing to the perpetuation of the undue importance of USMLE Step 1.

An undue emphasis on a single high stakes summative evaluation creates a dilemma for medical educators and students [73]. An excessive focus develops on preparing students for the USMLE Step 1 examination and “teaching to the test” [20, 74]. This milieu is counterproductive to in depth assimilation of subject matter in the biomedical sciences. Unintended consequences in multiple domains include conflict with holistic undergraduate medical education admission practices, student well-being, and medical curricula.

Medical students have become increasingly aware of the “USMLE issue.” In an Invited Commentary, medical students from various institutions across the country have reflected on their shared experiences and have postulated that the emphasis on USMLE Step 1 for residency selection has fundamentally altered the preclinical learning environment, creating a “Step 1 climate” [44]. They have commented on how the Step 1 climate negatively impacts education, diversity, and student well-being, and they have urged a national conversation on the elimination of reporting Step 1 numeric scores. Educators also have articulated similar recommendations regarding making the USMLE results reporting as pass/fail [75, 76]. But concern has also been voiced that pass/fail can be a disincentive to motivation for broad knowledge acquisition. Also, the development of an alternate, more holistic standardized metric by which to compare students’ applications for residency positions has been proposed but is currently not operative [74].

The movement away from meaningful grades for medical school courses also has led to an increasingly elaborate subjective evaluation in “dean’s letters” [77, 78]. The AAMC has introduced the Medical Student Performance Evaluation (MSPE) as a refinement of the “dean’s letter.” Approaches to evaluation of student performance generally involve formative and summative exams in the pre-clinical years, and subject exams coupled with faculty assessment of performance, in the clinical clerkships. Then, these evaluations (honors, high pass, pass, etc.) are integrated into lengthy MSPEs or dean’s letters that provide commentary and largely subjective impressions. In spite of the AAMC guidelines of comparative information about applicants be included, dean’s letters or MSPEs often continue to lack specificity regarding student performance [77, 78]. Major emphasis continues to rest on USMLE scores for the granting of interviews and ranking of applicants by residency program selection committees [74].

A second influential criterion relied upon in resident candidate ranking and selection is election to the Alpha Omega Alpha (AOA) Honor Society from the top one-sixth of the class. Election into AOA has long been a motivator for student performance. A relationship between AOA membership and selection into highly competitive residencies is well known [79]. AOA is receiving criticism that membership is not reflecting the balance of diversity of the student body [80, 81]. But, I hold that AOA must maintain a focus on excellence [82].

The grade abolition movement misses the reality of competition in human affairs. I think that the dilemmas about the “USMLE issue” can be diffused by a return to providing meaningful grades for medical school courses and an overall summative evaluation for the four years of medical school. (My definition of meaningful grades encompasses either numerical or letter grade equivalents which reflect actual performance relative to other students and objective norms.) Students must compete and excel to gain admittance into medical school. This shouldn’t be any different when students are training to be physicians. Safeguards can be put in place to deal with excess competition [33]. Nevertheless, competition within bounds promotes excellence. I strongly concur with the view that medicine is based on being a meritocracy and needs to remain a meritocracy [82, 83].

### Impact on medical educators

Over the years, medical educators, including basic biomedical science educators and clinician educators, have had to adapt to changes in curriculum [84–86]. Many medical educators have experienced significant challenges in the implementation of the new curriculum [87]. Competing demands on faculty time are causing stress and burnout among faculty as well as learners. A curriculum heavily geared to small group teaching places further considerable demand on faculty time. A significant inverse relationship has been found between faculty members’ readiness to change teaching approaches and their severity of burnout [87].

The educational mission itself can be enhanced by the recognition of foundational principles for teaching and education [88]. At Johns Hopkins University School of Medicine, a formal review process has led to the articulation of 10 foundational principles or characteristics of a medical educator [88]. Each principle addresses an important theme in the educational mission. These principles include specific recognition of the importance of being a role model and the responsibility to develop the next generation of physicians (Table 1).

**Table 1:** Foundational Principles of the Medical Educator

1. Embrace science and instill this passion in learners.	
2. Demonstrate integrity and thoroughness, and expect this from learners.	
3. Be a role model for honesty, integrity and kindness, and fair, equitable and respectful treatment of others.	
4. Instill in learners an appreciation for the importance of individual variability in human biology, genetics, behavior, and environment.	
5. Foster a positive learning environment that is diverse, respectful, inclusive and collegial.	
6. Develop the next generation.	
7. Always strive for excellence and aspire to continually do better.	
8. Teach and serve as a role model for the wise use of society’s resources.	
9. Help learners understand and appreciate the value of collaboration across disciplines.	
10. Demonstrate a focus on the public good.	

Adapted from (reference 88): Cofrancesco Jr. J, Ziegelstein RC, Hellmann DB. Developing foundational principles for teaching and education for a school of medicine. The Pharos/Spring 2018, pp. 43–46

### Ethics, professionalism and inter-professionalism in the curriculum

A major goal of the new curriculum is the development of holistic, ethical physicians with clear communication skills imbued with empathy and compassion for patients [29]. These goals are not new but are imbedded in the ideals of the medical profession which are intrinsic to its code of ethics [89]. There is a longstanding consensus that professionalism and professional identity formation need to be key elements of medical education [29]. However, a unifying theoretical or practical model to integrate the teaching of professionalism into the medical curriculum has not emerged [90, 91]. Nevertheless, role modeling and personal reflections -- ideally guided by faculty -- rather than blocks of time devoted to didactic exercises -- are widely held to be the most effective techniques for developing professionalism [90, 91]. Inter-professional education, another major contemporary thrust, also is best addressed after a foundation in the biomedical sciences is developed [92].

Regarding the issue of classroom attendance, medical student and teaching faculty attitudes have been found to differ regarding the importance of classroom attendance and its relationship to professionalism, findings that were at least partially explained by differing expectations of the purpose of the preclinical classroom experience [45]. Students tended to view class-going primarily as a tool for learning factual material, whereas many faculty viewed it as serving important functions in the professional socialization process [45]. Rather than dealing with practical solutions to enhancing the value of lectures, other formats are promoted which place inordinate demands on faculty time. This scenario is off-kilter. It sends the wrong messages to students regarding personal responsibility and professionalism. A practical approach to dealing with differing expectations and to effectively instill professionalism is to provide students, residents and staff with a written list of expected behaviors coupled with teaching and role modeling, assessment and remediation [93].

### Impact on pathology

Pathology is uniquely both a medical science and a clinical discipline [94–99]. In the analogy of the tree of medicine, the trunk is general pathology, which draws from all the basic biomedical sciences to elucidate general principles of regulation and dysregulation of homeostasis, and divides into the many branches of special pathology (organ system pathology); each one of these branches supports a specialized field of clinical medicine [95]. Thus, the place of pathology in the curriculum is seminally important in linking the basic biomedical sciences to clinical medicine and providing an understanding of the pathobiological basis of disease. The Association of Pathology Chairs has put forward a position paper on pathology competencies for medical education [99]. Since a solid understanding of pathology is core to the practice of medicine in any specialty, the position paper posits that all medical students must learn the basic mechanisms of disease, their manifestations in major organ systems, and how to apply that knowledge to clinical practice for diagnosis and management of patients. However, the place given to the pathobiological basis of disease in the new curriculum models is diminished.

Although a traditional curriculum includes a formal pathology course, pathology has been disadvantaged by the fact that students generally have little exposure to pathology or pathologists in the professionally formative clerkship years [100–102]. However, a distinction needs to be made between student perceptions of pathology as a career and pathology as a critically important medical science. The task of grounding medical students in principles of pathology, including pathogenesis and pathophysiology of disease, has been made considerably more difficult by the design of the new integrated, modular curriculum. The resultant discontinuance of pathology courses and their replacement by elements of pathology scattered episodically in the pre-clinical years likely has resulted in the dilution of core scientific principles and has contributed to a reduced understanding and interest in pathology [100–102].

Initiatives to increase the exposure and understanding of pathology and the autopsy are necessarily going to be tailored to the local environment operative at each institution [100–105]. While these approaches cannot fully substitute for the coherent presentation of the pathobiological basis of disease in a pathology course, it is imperative that pathology educators make this effort.

Nevertheless, exposure of medical students to the autopsy is a casualty of the current environment [106–109]. As a consequence, it is disconcerting but hardly surprising that physicians now in residency training and clinical practice have little understanding or appreciation for the autopsy, and, therefore, have little motivation for or experience with discussion of the autopsy with next of kin of the deceased. This state of affairs is contributing to the decline of the autopsy, which remains a uniquely important procedure for quality assurance in medicine [108, 109].

Another correlate of the current undergraduate medical educational environment is that pathology now has the lowest percentage of residency positions filled by U.S. seniors in the National Residency Matching Program [110, 111]. Furthermore, pathology residency programs have joined other medical specialties in conducting “boot camps” for incoming trainees [112–114]. The boot camps are aimed at providing the basics of a necessary foundation in pathology-specific medical science and in introducing basic skills and processes required for practice of anatomic pathology and laboratory medicine [112]. The assessment of pathology educators is that the new LCME-driven curriculum is producing a medical graduate who may *think* differently, but certainly lacks subject-specific knowledge for a variety of medical specialties. A putatively superior curriculum should not present a need for remedial learning for its graduates. Hopefully, boot camps for pathology trainees will be more effective than appears to be the case for bootcamps for trainees in surgical specialties [114].

### Impact on physician-scientists

Physician-scientists of various stripes have a unique and important role in translating basic science discoveries into advances in clinical medicine [115, 116]. Their numbers are small and their development is under threat. In some institutions, tailored curricula are being implemented to promote the development of clinician scientists [117, 118]. Nevertheless, there is a legitimate concern that the diminished position of basic science in the new curriculum is detrimental to the future maturation of physician-scientists [119].

Early predictors of career achievement in academic medicine have been identified as: 1) membership in AOA, 2) rank in the top third of the graduating class, and 3) research experience in medical school [9]. These three factors were of crucial importance in launching my career as was the seminal importance of a faculty mentor [120, 121]. The new curricula need to ensure that such opportunities are available for motivated medical students.

### Complexities and proposed solutions

Reformers contend that changes in the healthcare system and in medical practice in the clinic and hospital have outpaced those in the classroom, resulting in a declining relevance of the traditional curriculum and a growing urgency for a paradigm shift in medical education. Three barriers to the implementation of evidence-based curriculum reform have been identified [20]. First, curriculum revision must take place within a certain time frame, making it a zero-sum game. Second, transitioning from a few basic scientists lecturing entire classes from the podium to numerous small groups often tutored by clinical faculty dramatically increases the teaching demands on all faculty and especially faculty clinicians. Third, an inevitable tension is created between a holistic educational approach and the imperative to prepare students for USMLE Step 1.

Regarding the first point, reformers contend that reduction and revamping of the basic science content is warranted and can be achieved by elimination of perceived redundancy in the old curriculum. But the reality is that biomedical science, both in terms of curriculum time and emphasis, has been diminished in the new curricula [102, 118, 119]. Further negative pressure on the basic sciences is coming from the initiative to incorporate Health Systems Science into the curriculum with associated need to develop faculty with skills in teaching this material [23–27].

Pertinent to the second point, there are special challenges for faculty in educating the current generation of medical students in the Information Age [33, 40–42]. Certainly faculty educators need to recognize the characteristics of today’s students and take this into consideration in implementation of the curriculum. However, rather than taking a *laissez faire* approach, faculty educators need to set expectations regarding standards of performance [93]. In medical education, it is vital that faculty and staff temper overconfidence and excessive risk-taking [33]. Pedagogical approaches can be modified to meet the learning pattern of today’s medical students, for example, by blending lecture and non-lecture formats [43]. Nevertheless, standards for content and learning should remain the same; educators cannot compromise on the material that must be learned [33]. Also, medical students need to be taught and experience functioning and decision making in the face of inevitable uncertainties in life and medical practice [122, 123].

Regarding the third point, neo-curriculum advocates contend that solutions to the dilemma of the usurpation of the curriculum by the USMLE lie outside the control of undergraduate medical educators [20]. These advocates say that solutions require creativity and action from residency selection committees and the USMLE’s sponsors, the Federation of State Medical Boards and the National Board of Medical Examiners, because of the implementation of the new UME curriculum. But those in control of the UME curriculum can ensure that meaningful objective summative assessments of students in both pre-clinical and clinical courses are included in dean’s letters so that the USMLE is not the sole or primary objective assessment presented to residency selection committees.

In spite of the complexities, I contend that rebalancing the position of medical science in the medical educational curriculum has paramount importance [46–50, 102, 119]. This is to be achieved by providing the necessary amount of unencumbered space freed of major competing priorities. Different schools may use different approaches. Nevertheless, I favor restoration of subject-based courses, including a pathology course. Appropriate coordination of subject matter among the courses is essential, but this does not require the modular integration approach. Optimal ways of integrating topics in Health Systems Science during the multiyear curriculum need to be developed such as not to unduly compete with education in the core medical sciences.

### Trends in American healthcare, academic medical centers and academic medicine

Contemporaneous with restructuring of medical education, medical practice has undergone a fundamental transformation, dominated by a fixation on increasing efficiency in the delivery of care with quality of care a secondary consideration [124, 125]. The standard for the medical product has become good enough rather than excellent.

Regarding academic medicine, from 1985 to 2008, the percentage of active doctors engaged in teaching, research or administration decreased from 9 to 5.5%, and the number of teachers and mentors per US medical graduate declined from 0.11 to 0.07 [124, 125]. During the decade prior to 2004, biomedical research funding from all sources in America increased at an annual rate of 6.3%, and the United States funded more than half of all biomedical research conducted throughout the world. Since 2004, the growth rate for research funding has decreased to 0.8%, and the U.S.’ share of the world’s research investment has decreased to 44%. From 1996 to 2014, the percent of Nobel laureates in medicine or physiology who were at US institutions at the time of the award decreased from 80 to 45% [124, 125].

These very disturbing trends underscore some of the final words of the noted astrophysicist, Stephen Hawking, who warned that education and science around the world are “in danger now more than ever before” [126].

As eloquently stated by Brigham and Johns, the essence of excellence in medicine is more than doing what we know to do well, but must include a commitment to discovering what will make the better possible, and a dedication to perpetuating the best of the profession [125]. I content that countering the very disturbing trends just described is going to require a major multifaceted effort including a renewed commitment to advocacy for education and science and the rigorous education of new scientifically grounded physicians and physician-scientists who can carry the torch forward.

### The essence of a physician

As articulated over 100 years ago, the characteristics of the ideal physician extend to personal life, professional life and public life [127]. There is a broad consensus that the good doctor manifests a combination of humanistic and scientific attributes and capabilities [128, 129]. Seven key roles of the ideal doctor have been identified as communicator, collaborator, manager, health advocate, scholar, professional, and the integrating role of medical expert. Importantly all the roles overlap equally to create the ‘Medical Expert’ [130, 131]. Movement from novice to master in medicine (medical expert) cannot be rushed. Time, experience –and yes, repetition -- is necessary for maturation. This maturation needs to be built on a solid foundation in biomedical science and the pathobiology of disease. The time and place to inculcate the core of this foundation is the first two years of the UME. There are many years for learning and perfecting clinical skills and evidence-based medicine. This will not happen effectively without a sound foundation in biomedical science. A byproduct of a restoration of a strong medical science curriculum will be a boost to the development of future generations of physician-scientists. Conversely, the combination of educational deficiencies coupled with lifestyle preferences carries the risk of diminishing the status of future physicians [33].

## Conclusion

Enthusiasm for reform needs to be tempered by a more cautious and realistic approach. Unless there is modulation, the new curriculum is at risk of producing graduates deficient in the characteristic which have set physicians apart from other healthcare professionals, namely superior clinical expertise based on a deep grounding in biomedical science and understanding of the pathobiology of disease. Physicians need to remain the preeminent medical experts who strongly rely on understanding of basic mechanisms, particularly in dealing with difficult cases [47–49].

The overarching goal of medical education is the imparting of the highest principles, knowledge and skills in the nascent physician -- not bending medical education to follow prevalent but counterproductive personal and cultural trends. Our society requires physicians who will not just fit into the current dysfunctional American healthcare system but rather work to change it [11–14].

Medicine is a field that attracts people who want to have an impact, and this desire can be harnessed to improve medical education. The character traits of today’s medical students can potentially be harnessed to good ends, such as helping others through medicine. Good medical education resembles evolution in that it advances by ensuring the advancement of the fittest, including the fittest of the current generation of medical students just as the fittest of previous generations have succeeded in the past [33, 82, 83]. The challenges for education of the best possible physicians are great but the benefits for medicine and society are enormous.

Into the future, medical education Quo Vadis?

## Abbreviations

AAMC: American Association of Medical Colleges
ACGME: Accreditation Council for Graduate Medical Education
AHC: Academic health center
AMA: American Medical Association
AOA: Alpha omega alpha honor medical society
EPA: Entrustable professional activity
GME: Graduate medical education
LCME: Liaison Committee for Medical Education
MCAT: Medical college aptitude test
MSPE: Medical Student Performance Evaluation
NAS: New accreditation system
UME: Undergraduate medical education
USMLE: United States Medical Licensing Examination
